# Development of a nursing intervention to facilitate optimal antiretroviral-treatment taking among people living with HIV

**DOI:** 10.1186/1472-6963-9-113

**Published:** 2009-07-03

**Authors:** Pilar Ramirez-Garcia, José Côté

**Affiliations:** 1 Faculty of Nursing, Université de Montréal, Montréal, Québec, Canada; 2 Innovative Nursing Practice, CRCHUM, Faculty of Nursing, Université de Montréal, Montréal, Québec, Canada

## Abstract

**Background:**

Failure by a large portion of PLHIV to take optimally ARV treatment can have serious repercussions on their health. The absence of a systematic treatment-taking promotion program in Quebec prompted stakeholders to develop jointly a theory- and evidence-based nursing intervention to this end. This article describes the results of a collective effort by researchers, clinicians and PLHIV to share their knowledge and create an appropriate intervention.

**Methods:**

Intervention mapping was used as the framework for developing the intervention. First, the target population and environmental conditions were analyzed and a literature review conducted to identify predictors of optimal treatment taking. The predictors to emerge were self-efficacy and attitudes. Performance objectives were subsequently defined and crossed-referenced with the predictors to develop a matrix of change objectives. Then, theories of self-efficacy and persuasion (the predictors to emerge from step 1), together with practical strategies derived from these theories, were used to design the intervention. Finally, the sequence and content of the intervention activities were defined and organized, and the documentary material designed.

**Results:**

The intervention involves an intensive, personalized follow-up over four direct-contact sessions, each lasting 45–75 minutes. Individuals are engaged in a learning process that leads to the development of skills to motivate themselves to follow the therapeutic plan properly, to overcome situations that make taking the antiretroviral medication difficult, to cope with side-effects, to relate to people in their social circle, and to deal with health professionals.

**Conclusion:**

The intervention was validated by various health professionals and pre-tested with four PLHIV. Preliminary results support the suitability and viability of the intervention. A randomized trial is currently underway to verify the effectiveness of the intervention in promoting optimal antiretroviral treatment taking.

## Background

The effectiveness of antiretroviral (ARV) treatments in improving quality of life and life expectancy among people living with HIV/AIDS (PLHIV) has been demonstrated [1-3]. However, positive results are directly related to taking ARV treatments consistently and optimally, leading to total long-term viral suppression and, in turn, immunologic recovery [4-7]. Yet, in both clinical follow-ups and the scientific literature, a significant percentage of PLHIV have been reported not to persevere with optimal treatment taking [8,9]. In Quebec, a longitudinal study [10] showed that at each three-month follow-up 15% to 18% of participants had not taken their ARV treatment optimally in the past seven days. After one year of follow-up, only 50% of the participants continued to take the treatments at the optimum level at each time of measurement. Furthermore, at the first forum organized by COCQ-Sida (Quebec Coalition Against Aids) in 2004, PLHIV in the province described the challenges inherent in taking ARV treatment every day and expressed their need for assistance and support in order to adopt and maintain this behaviour. Against this backdrop, an intervention to facilitate optimal ARV-treatment taking would be of benefit to PLHIV in the community.

Scientific studies evaluating interventions to optimize ARV-treatment taking are a recent phenomenon, with most having been published since 2000 [11-14]. About 20 studies have demonstrated the capacity of certain interventions to promote treatment-taking behaviour, and five of these have also observed a clinical effect. Interventions in these randomized trials were administered to individuals with the intent of providing them with medication-management skills [15]. However, none of these interventions was based on a theoretical framework that provided a comprehensive understanding of the behaviour involved in taking an ARV treatment or laid down ways to foster this behaviour.

In the light of these findings and the fact that no systematic intervention existed for PLHIV in Quebec, a theory- and evidence-based intervention was deemed necessary to optimize ARV-treatment taking in the community. The purpose of this article is to document the results of a collective effort by researchers, clinicians and PLHIV to share and combine their knowledge in the aim of developing such an intervention.

## Methods: intervention mapping

We followed the guidelines proposed by Bartholomew, Parcel, Kok and Gottlieb [16,17] in a method referred to as "intervention mapping" (IM), a stepwise approach for the development and subsequent implementation of interventions grounded in theory and empirical data. It provides a framework that affords intervention planners a systematic mode for integrating theory, research results and field data collected from the target population at each phase of intervention development. Although we present intervention mapping as a series of six steps, the process is actually iterative and cumulative. Intervention developers base each step on the previous one, moving back and forth between tasks and steps as they gain information and different perspectives from various activities.

Accordingly, researchers must first conduct a needs assessment or problem analysis. This encompasses a review of the scientific literature to identify the main determinants of health behaviour as well as an analysis of the target population and environmental conditions. Second, researchers create matrices of change objectives based both on performance objectives and on predictors of optimal behaviour. Third, theoretical foundations must be defined and underlying theoretical methods and practical strategies identified. Fourth, researchers must translate methods and strategies into an organized intervention. Fifth, researchers must plan the adoption, implementation and sustainability of the intervention. Finally, an evaluation plan must be provided for.

### Step 1: Needs assessment

To gain a comprehensive understanding of the overall needs of the community of people on ARV treatment and to assess the capacity to modify their behaviour in this regard, we entered into a partnership with COCQ-Sida, a coalition of community organizations involved in the struggle against AIDS in Quebec. Our involvement in the coalition's "Treatment Adherence Committee" enabled us to collaborate with nurses, social workers, PLHIV and other stakeholders throughout the course of intervention development.

#### Analysis of target population and environmental conditions

The number of PLHIV in Quebec has been estimated at between 13,300 and 19,600 [18], with about two-thirds living in the health and social services region of Montreal [19]. Approximately 83.4% are males of Canadian origin and the majority has had homosexual relations.

In Quebec, the public healthcare insurance system ensures universal access to health services and treatment, including ARV medication. Clinical follow-up of PLHIV is ensured by general practitioners in family-medicine clinics and by microbiologists in outpatient clinics. Such follow-up is largely medical and focuses on the clinical facets of the infection. As well, there are community groups that provide information on and peer support for HIV and treatment. However, there is no guidance or systematic intervention to foster ARV-treatment taking this population.

#### Analysis of behaviour

To promote optimal ARV-treatment taking, it is important to understand the behaviour and identify the factors associated with it. In their review of the literature, Ramirez-Garcia and Côté [20] found the most significant modifiable factors in this regard to be social support [21,22], attitudes [23], self-efficacy [24,25], frequency of treatment regimen [26], side-effects [27,28] and degree of satisfaction with health professionals [23,29].

A longitudinal study of 376 PLHIV in the target community [10] confirmed the importance of treatment taking and the influence of the above factors. The best predictive model included high self-efficacy, positive attitudes toward taking medication, not living alone and being male. Secondary analysis demonstrated that, of the above factors, the ones that could be improved so as to affect self-efficacy were social support and satisfaction with healthcare professionals. The same two factors and the absence of adverse side-effects also influenced attitudes. Thus, the theoretical framework of Godin et al. [10] was not only useful in predicting optimal medication-taking behaviour but also in explaining how such behaviour could be fostered in PLHIV.

The needs assessment culminated with the definition of the ultimate objective of the intervention, the desired behavioural outcome. For this project, it would be optimal ARV-treatment taking among PLHIV in the community.

### Step 2: Matrix of intervention objectives

Objectives were further specified at this step of IM. First, the behavioural outcome was broken down into its component parts. These components parts were referred to as a performance objective. For this project, we formulated five performance objectives: follow treatment plan properly as part of daily routine, overcome situations that make taking medication difficult, cope with side-effects, relate well to those in one's personal social circle, and interact effectively with health professionals.

Finally, we formulated change objectives. These were the most immediate goals for the intervention to accomplish and were stated in terms of exactly what a person needed to learn to facilitate performing a specific behaviour. Change objectives were formulated by examining the performance objectives with regards to the predictors of optimal ARV-treatment-taking behaviour. Table 1 presents the matrix of intervention objectives for two performance objectives.

**Table 1:** Matrix of intervention objectives

**Performance objectives**	**Predictors**	
	**Self-efficacy**	**Attitudes**
PO.1. Overcome situations that make taking ARV medication difficult	SE.1a. Identify negative emotions in these situations: record and describe negative emotions in these situations	A.1a. Anticipate negative consequences of negative emotions on behaviour
	SE.1b. Express confidence that one can cope with one's emotions	A.1b. Expect to avoid and cease negative emotions
	SE.1c. Use (entertainment or relaxation) strategies to avoid and cease negative emotions	A.1c. Argue that negative emotions can be avoided or ceased
	SE.1d. Identify patterns or situations that make taking medication difficult	
	SE.1e. Express confidence that one can take medication in every situation	A.1e. Argue that, in dealing with situations that make taking medication difficult, there will be fewer omissions
	SE.1f. Adopt new tools or strategies to overcome difficult situation and take ARV medication	A.1f. Expect to deal with situations that make taking medication difficult and fewer omissions in future
PO.2. Deal with health professionals	SE.2a. Identify role of healthcare professionals with regard to ARV therapy SE.2b. Recognize situations that call for advice from various health professionals	
	SE.2c. Express confidence that one can ask for advice from various health professionals about ARV therapy	A.2c. Consider healthcare professionals as health partners or councillors who can provide means to take ARV tretament
	SE.2d. Correctly use strategies to ask for advice from health professionals about ARV-treatment taking	A.2d. Expect that interacting with various health professionals will be beneficial to optimizing ARV-medication taking

### Step 3: Theoretical methods and practical strategies

Step 3 in IM consisted in selecting useful theory-based methods to influence behaviour and the strategies to organize and operationalize the intervention methods. Two theories served as the foundation for the development of our intervention: Bandura's self-efficacy theory [30] and Petty and Cacioppo's persuasion theory for attitudinal change [31].

#### Self-efficacy theory

Bandura defined self-efficacy as an individual's belief in his or her own ability to organize and implement action to produce a desired result. He stated that individuals must have firm confidence in their abilities to undertake and follow through with the procedures necessary to succeed. He argued that individuals took action when a sense of efficacy made the effort seem worthwhile in order to attain the expected results. He further defined result expectations as opinions about the probable outcomes that actions would lead to and considered an individual's attitude to be a form of these expectations.

According to Bandura [30], self-efficacy is a productive characteristic that is crucial to acquiring the skills necessary to adopt a behaviour. It affects cognitive, motivational and emotional processes, and these can improve or deter performance. In view of this, skills acquisition in these areas helps not only establish behaviour considered necessary but also control the influence of these processes on learning and on behaviour. Finally, Bandura argued that self-efficacy was a contributor to the acquisition of these skills in that it relied on them to promote desired behaviour. In this intervention, four principal sources described by Bandura were used to enhance self-efficacy: sense of mastery, vicarious experiences, verbal persuasion, and management of physiological and emotional states.

According to self-efficacy theory, "mastery experiences" are the most influential means of expanding one's efficacy because they show most clearly how people can bring together the things they need to succeed. Bandura considered that mastering difficult tasks afforded people a new perspective on efficacy by increasing their confidence in their own capabilities. He regarded problem solving as a cognitive skill that facilitated learning how to master difficult tasks. For starters, he believed that the individual must learn to monitor his or her behaviour to identify conditions in which the behaviour arose. In his view, self-observation was a self-regulating motivational skill that was necessary to be able to set realistic objectives, evaluate progress and, as a result, nurture a sense of personal efficacy.

With regard to "vicarious experience," Bandura stated that evaluations of efficacy were partly influenced by the achievements of others. The more an individual identified with these others, the more influential their experiences of success became. He suggested models that taught these skills could stimulate the personal efficacy of individuals. As well, individuals could draw a greater benefit from examples in which difficulties were overcome by perseverance rather than from examples of easier tasks carried out by expert models. For Bandura, a cognitive model or visualization was a vicarious experience that influenced subsequent performance. Our intervention incorporated visualization into the problem-solving process.

Bandura [30] maintained that individuals verbally "persuaded" of having the skills to master certain activities were more likely to make an added effort and sustain it than self-doubting individuals were. He asserted that such verbal persuasion, which highlighted personal abilities and progress, boosted self-efficacy. He saw information about other peoples' success norms and the attribution of failure to either ineffective strategies or degree of task difficulty as persuasion strategies that strengthened the sense of self-efficacy.

Moreover, Bandura stated that people often interpreted their physiological and emotional reactions to stressful or demanding situations as signs of vulnerability or dysfunction. He believed that the skills to control these emotional reactions heightened the sense of efficacy, resulting in a corresponding improvement in performance. Hence, we included the acquisition of emotional self-management skills in our intervention.

**Figure 1:**
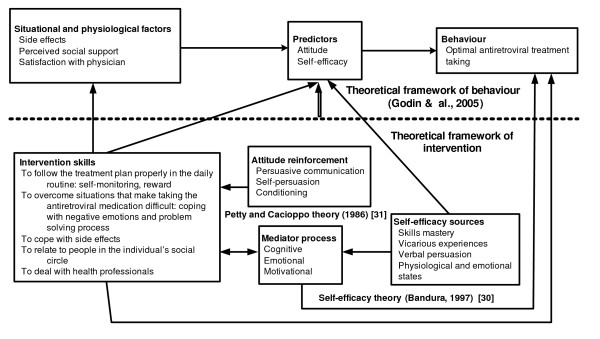
**Theoretical structure of intervention**.

The theoretical structure of or intervention is presented in diagram form in Figure 1. The diagram shows the matrix of relationships between the empirical basis of behaviour, such as the predictors of ARV-treatment-taking, and the theoretical basis, which recommends which skills should be developed to facilitate this behaviour.

#### Persuasion theory

Under the Elaboration Likelihood Model proposed by Petty and Cacioppo [31], persuasive communication is seen as central to bringing about attitudinal change. The term "elaboration" refers to the extent to which individuals receiving a message will devote an effort to pondering its contents in shaping their attitudes. These authors describe two courses for processing information: central and peripheral.

When individuals are motivated and able to think and understand the message, the model predicts that they will follow the central course to make up their minds. However, when an individual is distraught or unmotivated, the information will be processed peripherally. Petty and Cacioppo [31] consider attitudes formed or changed via the central course to be relatively enduring predictors of behaviour and resistant to change. Consequently, this is the course that should be favoured for attitude reinforcement or change. To ensure the effectiveness of a persuasive message, it should therefore be framed in substantive terms and conveyed when individuals are motivated and able to concentrate on it.

When individuals are distressed or unmotivated, other peripheral mechanisms, such as the cognitive-reasoning process, the affective-conditioning process and social influence should be applied. Similarly, Petty, Wegener and Fabrigar [32,33] observed that a simple means of influencing people's attitudes in either a positive or negative direction was to ask them to analyze the positive or negative aspects of the behaviour. This cognitive process is known as self-persuasion. The authors added that repeatedly associating behaviour with positive feelings or with a stimulus that produced positive feelings (i.e., an affective "conditioning" process) could lead to positive attitudes toward the behaviour. Both of these methods were incorporated in our intervention (Table 2).

**Table 2:** Theories, methods and strategies regarding targeted predictors of behaviour

**Predictors/Theories**	**Methods**	**Strategies**
**Attitudes** (Petty and Cacioppo, 1986 [31]; Petty, Wheeler and Fabrigar, 1997 [33]; Petty, Fabrigar and Wegener, 2003 [32])	Persuasive communication	Tailored information Explanation/reflection or discussion Constructive feedback on positive attitudes
	Self-persuasion	Reflection or discussion
	Conditioning	Reward after behaviour Association of positive thoughts
**Self-efficacy/skills** (Bandura, 1997) [30]	Direct experience of mastering issues	Self-observation of behaviour: recognition of success Problem-solving process Establishing goals Application of skills/constructive feedback
	Vicarious experiences	Modeling: Case history Self-modeling or visualization
	Verbal persuasion	Attribution of cause of mistakes to inadequate strategies or difficulty of task Allusive feedback (comparison to other groups or norm) Constructive feedback on acquisition of skills
	Physiological and emotional state	Demonstration of impact of negative thoughts on behaviour Explanation/reflection or discussion on activating emotions Application of emotion-management skills

### Step 4: Intervention

At this step, the intervention was designed by translating the theoretical methods and practical strategies into intervention documents. This intervention drew its inspiration from a philosophy of empowerment. In this approach, the PLHIV must be actively involved in seeking the information required to foster optimal ARV-treatment taking behaviour, and the health professional becomes a partner in care and a counsellor helping the individual develop the skills to facilitate this behaviour.

The intervention consisted of a customized follow-up of four 45- to 75-minute sessions with an HIV-expert nurse. The first three sessions focused on the appropriation and mobilization of specific skills in a planned order: motivational skills to follow the therapeutic plan properly and skills to cope with side-effects, including skills to identify and manage them (first session); skills for coping with negative emotions and for problem-solving to deal with situations that make taking ARV medication difficult (second session); skills for relating to people in one's social circle and for dealing with health professionals (third session). The skills thus learned were boosted in the final session.

While each session focused on specific skills, it was always possible to review those discussed previously. Different tools were utilized to facilitate use of the skills acquired, such as a chart for observing behaviours and a personal diary for recording side-effects (developed by the principal investigator and validated by HIV-expert health professionals). The individuals' understanding and use of the new skills were evaluated and positively reinforced at each session. As well, at the end of each session, the nurse suggested that the individual set objectives for the next one. These objectives would guide and motivate the individuals to integrate the skills they had learned in their daily routines and to verify their usefulness [30]. Various documents were used to assure retention of the new information, for example, leaflets on the range of ARV treatments available [34] and leaflets recommending ways of dealing with adverse side-effects. Specifically, each meeting was organized and proceeded as follows.

#### First session

During the first session the individual was called upon to develop and reinforce skills that would be useful in taking ARV medication as part of the daily routine. For starters, the individual was given personalized explanations on how the treatments worked, their purpose, the importance of taking them (strictly following a regular regimen), how to take them (with or without food, intervals, storing the product properly) in addition to tools and strategies to make taking medication easier (pillbox, clock radio or alarm). This educational component paved the way for the development of skills and was tailored to the individual's needs and level of awareness. This session was also designed to help develop motivational skills, such as monitoring and rewarding behaviours. Each of these was supposed to foster ARV-treatment taking.

One strategy for developing a positive attitude toward taking ARV medication was to associate each dose with a positive image or message, such as that of the sea or the sun or repeating that the medication would preserve good health longer. Assessing one's own behaviour (self-regulating skills) involved analyzing circumstances and the situation in which treatment was neglected or forgotten: "Who was I with? What was I doing?" This process would lead to finding ways to resolve the problem. Conversely, the individual analyzed situations in which he or she successfully took the medication and gained the incentive to do so again. Attaching positive significance to treatment taking would make taking medication repeatedly with a positive attitude all the easier. The final objective of the session was to enable individuals to identify and deal with adverse side-effects of the ARV treatment. With the nurse's help the side-effects were identified, as were ways of avoiding or moderating them.

#### Second session

The second session focused on skills to cope with negative emotions and resolve problems. As negative attitudes deter motivation and affect behaviour, they have detrimental repercussions on ARV-treatment taking. It was therefore important to be able to recognize them and keep them from persisting or intensifying. The most effective means of managing thoughts and feelings were discussed; most notably, individuals learned to repeat calming, encouraging and comforting phrases (positive messages) to themselves [30]. As well, techniques for self-distraction, such as reading, watching films and seeking comfort from companions were reinforced.

The problem-solving process was addressed in the second part of this session to equip the individual to overcome difficult or awkward situations. This process, adapted from Sharma, Petosa and Heaney [35] and from Lorig and Fries [36], consists in: 1) **d**escribing the situation in which the oversight occurred; 2) **e**laborating a list of strategies for dealing with the situation; 3) **c**hoosing the strategy with the highest probability of being useful; 4) **i**magining oneself facing up to the situation; 5) **d**eciding to take action and confronting the situation using this strategy; and 6) **e**valuating the results.

The process was presented by means of a case history, in the aim of facilitating learning through vicarious experience. It was then put into practice by the individual in a situation in which he or she found it difficult to take ARV treatment. A mnemonic technique using the first letter of each step of the process to spell out "**Decide" **made it easier to remember.

#### Third session

In this session, the focus was on mastering social skills and the skills for dealing with health professionals in order to enable individuals to mobilize formal and informal networks to assist with ARV-medication taking. Here, the nurse explained a process framed by Bergeron [37] that entailed identifying people likely to provide support and then establishing, reinforcing and maintaining relations with them. Thus, the individual could seek help and mobilize support.

This session began with an explanation of different types of social support (emotional, material, informational, and social integration), their importance and the impact they had on ARV-treatment taking. Next, the nurse asked the individual to think about which members of his or her social circle helped or could help with medication taking. The nurse then provided details of different community organizations involved in the struggle against AIDS and the services they offered. Strategies for creating opportunities to meet with other people, increase interaction, maintain contact and open dialogue with them were then discussed [38,39]. Two key communications skills – attentive listening and expressing feelings – were explained and demonstrated with concrete examples from daily life [40]. The nurse then used role playing to simulate seeking aid or mobilizing social support: This activity involved making a request, listening to the other person's reaction and giving feedback on the response or support received.

In the final portion of this encounter, the nurse explained the different roles of the health professionals involved (nurse, physician, pharmacist, dietician, psychologist, and sexologist) and described situations in which their help was called for. The nurse then went over the above communication skills again to demonstrate how to deal with professionals efficiently, which entailed preparing for meetings, listening carefully when taking in information, giving and receiving feedback, and obtaining responses to questions.

#### Fourth session

A final booster session was held to reinforce the skills that had been imparted. The nurse assessed whether the objectives had been attained and offered the individual the opportunity to work further on the skills covered in previous sessions.

Details of the intervention and copies of intervention materials are available on request.

### Step 5: Implementation

At this stage, it was important to ensure that the project met the needs of the target community, could be implemented and could obtain the necessary support of partners and decision-makers. The fact that this was the first time a systematic intervention to help with ARV-treatment taking had been offered had to be taken into consideration in planning how to implement it. It was important therefore to find out to what extent our designed intervention met the needs of PLHIV and was accepted by them. To this end, the content of the intervention and the documentation prepared for the participants were validated by HIV health experts: two nurses, two dieticians, a physician and a pharmacist. They used a four-point Likert-type scale to evaluate the accuracy, clarity, relevance, and importance of the information provided in the intervention and the documentation to be given to the participants. They also expressed opinions on the relevance, importance, feasibility, and acceptability of the intervention and were asked to include explanations or justifications whenever they rated items on the scale as having little or no relevance, importance, accuracy, or clarity (i.e., 1 or 2) or when they had other observations about the intervention. Generally speaking, these professionals gave the pertinence of the information provided in both the intervention and the documentation a rating of 3 or 4. Information on CD4 count and viral load, the importance of taking medication appropriately and getting around side-effects received a rating of 2 for its precision and clarity. Accordingly, we re-formulated this material with the aid of the professionals' comments (some examples are given in Table 3).

**Table 3:** Validation comments by HIV health experts and PLHIV

**Sections/Topic**	**Observations made by HIV health experts**
Implications of CD4 count and viral load	"To avoid confusion, I suggest always referring to the CD4 count in terms of a decrease, rather than saying that CD4 should remain 'above' 350 or that the risk of opportunistic infections 'increases' if it falls below 350." "Explain RNA... For example: each virus has a similar genetic code, which is the virus RNA and which is identifiable through laboratory analysis... Each RNA = 1 virus." "According to DHHS guidelines, treatment is recommended if the CD4 count is between 200 and 350, regardless of the viral load ... and should be altered if the viral load is >100,000 and particularly if the viral load is very high."
Importance of taking medication effectively	"More and more people are taking treatment only once a day, so the significance of this should be stressed (in terms of missed dosages per month)." "Add more emphasis and explanation about mutations; for example: some of these errors allow the virus to become resistant to treatment... the virus can then multiply increasingly and the viral load increases again."
Techniques for contending with side-effects	"I advise seeking a consultation if a cutaneous eruption appears right away because there is a lot of syphilis around and it presents with a skin rash." "Add that it is important to consult a dietician when there is weight loss of more than 5% of the normal weight." "Instead of recommending avoiding or cutting out dairy product consumption, you could suggest alternatives such as trying lactase." "Instead of just saying that it is necessary to re-hydrate, be more precise about how to do this: prepare and drink pharmacy re-hydration beverages such as *Pédialyte^® ^*or *Gastrolyte^® ^*or make their own."
**Sections/Topic**	**Remarks made by PLHIV**
Importance of taking medication effectively	"I didn't understand the resistance part, it was difficult to follow; maybe you could add a diagram (sketch) to help us understand it." "I couldn't follow the material on mutations and resistance... It's all too complicated."
Recognizing oversights	"I thought I had understood, but when I got home and I wanted to make notes, I didn't know how to set about it: there should be more explanations and examples given.
Techniques for solving problems	"It's difficult to keep in mind all the steps: a written version of part should be available."

Furthermore, both the documentation and the intervention were pre-tested with four HIV-positive individuals, three men and one woman, on ARV medication. This was done to ensure that the intervention was acceptable and met the needs of PLHIV and that the language was clear with difficult and complex terms explained. They used the same Likert-type scale as the health professionals and provided comments and criticisms. These PLHIV gave the importance of the information in the intervention and the documentation a rating of 3 or 4. The small portion of the content that was rated 2 has since been modified (Table 3).

In general, all of the professionals gave the relevance, importance and suitability of the intervention and its feasibility a rating of 3 or 4. Their consensus was that the intervention was acceptable and that it was important to implement an intervention that could both foster ARV-treatment taking and that was consistent with the nature of the community. Similarly, the PLHIV expressed their satisfaction with the intervention and with the new skills it afforded them.

In order to obtain support for setting it up the intervention, COCQ-Sida assisted in contacting a family medicine clinic specialized in sexually transmitted diseases (STDs) and HIV/AIDS in Montréal. A service agreement was reached with the clinic to make our intervention available to PLHIV, in exchange for physical space for carrying it out. To facilitate cooperation with the clinic's professionals in identifying and referring participants, a number of presentations were made on the content of the intervention. A fundamental aspect of this project was its implementation by the researcher/clinician, who was present full-time in the clinic to facilitate its integration by the team of health professionals and to assist with participant recruitment.

### Step 6: Evaluation plan

An experimental design was developed to evaluate the effects of the intervention. The intensive personalized follow-up over four direct-contact sessions would be compared with usual care. The usual-care intervention was not systematic. It was performed by the clinic nurse and consisted of a detailed explanation of the prescribed treatment and follow-up at the request of the PLHIV. The following hypotheses were tested: a greater proportion of participants receiving the intervention than of those in the control group would have taken their medication optimally at the T3- and T6-month follow-ups. Self-efficacy, attitudes, side-effects, social support, and relations with health professionals were mediator variables correlated with optimal treatment taking. Optimal treatment taking, the principal outcome, was evaluated on the basis of a self-administered questionnaire, viral load and CD4 count. The results of this evaluation will be published soon.

## Discussion

The importance of behaviour and the factors that determine it in the achievement of optimal ARV-treatment taking is widely recognized. Few studies, however, have demonstrated the effectiveness of interventions to promote optimal medication taking, in part, however, owing to the poor methodology used [14,15]. There are also gaps in the information on the development of interventions. Apparently, they have not always been founded on a solid theoretical or empirical framework and are largely derived from components of information. This information content is clearly essential, but it is not sufficient to facilitate optimal treatment taking [41].

Nonetheless, the authors of a recent exhaustive review of the literature [15] did find evidence supporting the effectiveness of some interventions in this regard. In fact, they were able to identify certain criteria for effectiveness: The interventions have to target medication-management skills and must be administered to individuals rather than to groups, and the duration of the intervention has to be at least 12 weeks. Results of other randomized trials carried out since the literature review in question also support these conclusions [42-44].

These findings justify our efforts to develop a solid theory-based nursing intervention that enables us to explain and predict behaviour that fosters optimal ARV-treatment taking. Using a systematic approach that allowed the integration of a theoretical framework, we structured our intervention on the basis of empirical and clinical data. This interweaving of different knowledge sources is an obvious strength of the mapping approach: It allows the proficiencies of all participants (researchers, clinicians and PLHIV) to converge in the development of interventions. Furthermore, we incorporated the three criteria of efficacy identified by Rueda and colleagues [15].

There remains only to verify the efficacy of this intervention in optimizing ARV-medication-taking behaviour. To this end, a randomized was realized. We believe that an intervention based on a solid theoretical framework aimed at the acquisition of skills necessary to manage ARV-medication will foster optimal ARV-treatment taking behaviour among PLHIV.

## Conclusion

This article describes the results of a collective effort by researchers, clinicians and PLHIV to share their knowledge and create an intervention to promote optimal ARV-treatment taking. Intervention mapping was used as a framework for developing the intervention. The goal of the intervention was the acquisition and mobilization of skills to manage ARV-medication taking and the enhancement of self-efficacy and positive attitudes towards ARV-treatment taking, thus foster the desired behaviour.

## Competing interests

The authors declare that they have no competing interests.

## Authors' contributions

PRG and JC conceived and developed the intervention. PRG drafted the manuscript. All authors read and approved the final manuscript.

## Pre-publication history

The pre-publication history for this paper can be accessed here:



## References

[B1] Casalino E, Wolff M, Ravaud P, Choquet C, Bruneel F, Regnier B (2004). Impact of HAART advent on admission patterns and survival in HIV-infected patients admitted to an intensive care unit. AIDS.

[B2] Hogg RS, Yip BKC, Craib KJP, O'Shaughnessy MV, Schechter MT, Montaner JSG (1999). Improved survival among HIV-infected patients after initiation of triple-drug antiretroviral regimens. CMAJ.

[B3] Sterne JAC, Hernan MA, Ledergerber B, Tilling K, Sendi P, Rickenbach M, Robins JM, Egger M, Study tSHC (2005). Long-term effectiveness of potent antiretroviral therapy in preventing AIDS and death: a prospective cohort study. Lancet.

[B4] Bangsberg DR, Charlebois ED, Grant RM, Holodniy M, Deeks SG, Perry S, Conroy KN, Clark R, Guzman D, Zolopa A (2003). High levels of adherence do not prevent accumulation of HIV drug resistance mutations. AIDS.

[B5] Gardner EM, Sharma S, G P, Huppler-Hullsiek K, Burman WJ, MacArthur RD, Chesney M, Telzak EE, Friedland G, Mannheimer SB (2008). Differential adherence to combination antiretroviral therapy is associated with virological failure with resistance. AIDS.

[B6] Paterson D, Swindells S, Mohr J, Brester M, Vergis E, Squier C, Wagener MM, Singh N (2000). Adherence to protease inhibitor therapy and outcomes in patients with HIV infection. Ann Intern Med.

[B7] Sungkanuparph S, Groger RK, Overton ET, Fraser VJ, Powderly WG (2006). Persistent low-level viraemia and virological failure in HIV-1-infected patients treated with highly active antiretroviral therapy. HIV Medicine.

[B8] Delpierre C, Delmas P, Delon S (2006). Facteurs protecteurs et fragilisants de l'adhérence aux traitements des patients vivants avec le VIH: cas de la cohorte PROMOSUD [Protective and risk factors in treatment adherence by PLHIV: The case of the PROMOSUD cohort]. 74ème Congrès ACFAS «Le savoir trame de la modernité»: 15–19 May 2006; Montréal.

[B9] LeMoing V, Chêne G, Leport C, Lewden C, Duran S, Garré M, Masquelier B, Dupon M, Raffi F, group tACAs (2002). Impact of discontinuation of initial protease inhibitor therapy on further virological response in a cohort of human immunodeficiency virus-infected patients. Clin Infect Dis.

[B10] Godin G, Côté J, Naccache H, Lambert LD, Trottier S (2005). Predictors of adherence to antiretroviral therapy: a one year longitudinal study. AIDS Care.

[B11] Andrews L, Friedland G (2000). Progress in HIV therapeutics and the challenges of adherence to antiretroviral therapy. Infect Dis Clin North Am.

[B12] Fogarty L, Roter D, Larson S, Burke J, Gillespie J, Levy R (2002). Patient adherence to HIV medication regimens: a review of published and abstract reports. Patient Educ Couns.

[B13] Haddad M, Inch C, Glazier RH, Wilkins AL, Urbshott GB, Bayoumi A, Rourke S (2000). Patient support and education for promoting adherence to highly active antiretroviral therapy for HIV/AIDS. The Cochrane Database of Systematic Reviews.

[B14] Simoni JM, Frick PA, Pantalone DW, Turner BJ (2003). Antiretroviral adherence interventions: a review of current literature and ongoing studies. Topics in HIV medicine.

[B15] Rueda S, Park-Wyllie LY, Bayoumi AM, Tynan AM, Antoniou TA, Rourke SB, Glazier RH (2006). Patient support and education for promoting adherence to highly active antiretroviral therapy for HIV/AIDS. Cochrane Database Syst Rev.

[B16] Bartholomew LK, Parcel GS, Kok G, Gottlieb NH (2001). Intervention mapping: Designing theory- and evidence-based health promotion programs.

[B17] Bartholomew LK, Parcel GS, Kok G, Gottlieb NH (2006). Planning health promotion programs: An intervention mapping approach.

[B18] Actualités en épidémiologie sur le VIH/sida [HIV/AIDS EPI updates]. http://www.phac-aspc.gc.ca/publicat/epiu-aepi/epi-1205/index_f.html.

[B19] Programme de surveillance de l'infection par le virus de l'immunodéficiencia humaine (VIH) au Québec. Cas déclarés de janvier à juin 2006 et cas cumulatifs d'avril 2002 à juin 2006 [Program for monitoring HIV infection in Quebec: Declared cases from January to June 2006 and cumulative cases from April 2002 to June 2006]. http://www.inspq.qc.ca/publications/notice.asp?E=p&NumPublication=614.

[B20] Ramirez P, Côté J (2003). Factors affecting adherence to antiretroviral therapy in people living with HIV/AIDS. J Assoc Nurses AIDS Care.

[B21] Gordillo V, del Amo J, Soriano V, Gonzalez-Lahoz J (1999). Sociodemographic and psychological variables influencing adherence to antiretroviral therapy. AIDS.

[B22] Spire B, Duran S, Souville M, Leport C, Raffi F, Moatti JP, the APROCO cohort study group (2002). Adherence to highly active antiretroviral therapies (HAART) in HIV-infected patients: from a predictive to a dynamic approach. Soc Sci Med.

[B23] Stall R, Hoff C, Coates TJ, Paul J, Phillips KA, Ekstrand M, Kegeles S, Catania J, Daigle D, Diaz R (1996). Decisions to get HIV tested and to accept antiretroviral therapies among gay/bisexual men: Implications for secondary prevention efforts. J Acquir Immune Defic Syndr Hum Retrovirol.

[B24] Johnson MO, Catz SL, Remien RH, Rotheram-Borus MJ, Morin SF, Charlebois E, Gore-Felton C, Goldsten RB, Wolfe H, Lightfoot M (2003). Theory-guided, empirically supported avenues for intervention on HIV medication nonadherence: Findings from the Healthy Living Project. AIDS Patient Care and STD's.

[B25] Tuldra A, Fumaz CR, Ferrer MJ, Bayés R, Arno A, Balagué M, Bonjoch A, Jou A, Negredo E, Parades R (2000). Prospective randomized two-arm controlled study to determine the efficacy of a specific intervention to improve long-term adherence to highly active antiretroviral therapy. J Acquir Immune Defic Syndr.

[B26] Golin CE, Liu H, Hays RD, Miller LG, Beck CK, Ickovics J, Kaplan AH, Wenger NS (2002). Prospective study of predictors of adherence to combination antiretroviral medication. J Gen Intern Med.

[B27] Duran S, Spire B, Raffi F, Walter V, Bouhour D, Journot V, Cailleton V, Leport C, Moatti JP, group tAcs (2001). Self-reported symptoms after initiation of a protease inhibitor in HIV-infected patients and their impact on adherence to HAART. HIV Clinical Trials.

[B28] Roca B, Gomez CJ, Arnedo A (2000). Adherence, side effects and efficacy of Stavudine plus Lamivudine plus Nelfinavir in treatment-experienced HIV-infected patients. J Infect.

[B29] Bakken S, Holzemer WL, Brown MA, Powell-Cope GM, Turner JG, Inouye J, Nokes KM, Corless I (2000). Relationships between perception of engagement with health care provider and demographic characteristics, health status and adherence to therapeutic regimen in persons with HIV/AIDS. Aids Patient Care STDS.

[B30] Bandura A (1997). Self-efficacy: The exercice of control.

[B31] Petty RE, Cacioppo JT (1986). Communication and persuasion: Central and peripheral routes to attitude change.

[B32] Petty RE, Fabrigar LR, Wegener DT, Davidson RJ, Scherer KR, Goldsmith HH (2003). Emotional factors in attitudes and persuasion. Handbook of affective sciences.

[B33] Petty RE, Wegener DT, Fabrigar LR (1997). Attitudes and attitude change. Annu Rev Psychol.

[B34] Infection au VIH: Guide thérapeutique [HIV infection: A therapeutic guide]. http://www.jag.on.ca/hiv.

[B35] Shrama M, Petosa R, Heaney CA (1999). Evaluation of a brief intervention based on social cognitive theory to develop problem-solving skills among sixth-grade children. Health Educ Behav.

[B36] Lorig K, Fries JF (1990). The arthritis helpbook: a tested self-management program for coping with your arthritis.

[B37] Bergeron R (1994). Élaboration et évaluation d'une intervention infirmière de renforcement du soutien social auprès d'aidantes de parents atteints de démence. [Development and evaluation of a nursing intervention to reinforce social support for female family caregivers of a relative with dementia].

[B38] Cutrona CE, Cole V, Cohen S, Underwood LG, Gottlieb BH (2002). Optimizing support in the natural network. Social Support Measurement and Intervention.

[B39] Heaney CA, Israel BA, Glanz K, Rimer BK, Lewis FM (2002). Social networks and social support. Health behavior and health education: Theory, research and practice.

[B40] DeVito JA, Chassé G, Vezeau C (2001). La communication interpersonnelle [Interpersonal communication].

[B41] Côté J, Godin G (2005). Efficacy of interventions in improving adherence to antiretroviral therapy. Int J STD AIDS.

[B42] Chiou PY, Kuo BI, Lee MB, Chen YM, Chuang P, Lin PC (2006). A program of symptom management for improving quality of life and drug adherence. J Adv Nurs.

[B43] Holzemer WL, Bakken S, Portillo CJ, Grimes R, Welch J, Wantland D, Mullan JT (2006). Testing a nurse-tailored HIV medication adherence intervention. Nurs Res.

[B44] Williams BA, Fennie PK, Bova AC, Burgess DJ, Danvers AK, Dieckhaus DK (2006). Home visits to improve adherence to highly active antiretroviral therapy: a randomized controlled trial. J Acquir Immune Defic Syndr.

